# ERGA-BGE reference genome of Hanak's bat ( *Pipistrellus hanaki*), an IUCN Vulnerable species restricted to forest-like biotopes

**DOI:** 10.12688/openreseurope.20937.2

**Published:** 2025-11-11

**Authors:** Panagiotis Georgakakis, Danae Karakasi, Petros Lymberakis, Manolis Papadimitrakis, Manos Stratakis, Eleftherios Bitzilekis, Nikolaos Poulakakis, Astrid Böhne, Rita Monteiro, Rosa Fernández, Nuria Escudero, Genoscope Sequencing Team, Manon Angel, Jean-Marc Barbance, Julie Batisse, Odette Beluche, Laurie Bertrand, Elodie Brun, Maria Dubois, Corinne Dumont, Barbara Estrada, Thomas Guerin, Zineb El Hajji, Sandrine Lebled, Patricia Lenoble, Claudine Louesse, Ghislaine Magdelenat, Eric Mahieu, Claire Milani, Sophie Oztas, Marine Paillard, Emilie Payen, Emanuelle Petit, Murielle Ronsin, Benoit Vacherie, Alice Moussy, Corinne Cruaud, Karine Labadie, Lola Demirdjian, Sophie Mangenot, Caroline Belser, Patrick Wincker, Pedro H. Oliveira, Jean-Marc Aury, Leanne Haggerty, Swati Sinha, Fergal Martin, Chiara Bortoluzzi

**Affiliations:** 1 Natural History Museum of Crete, School of Sciences and Engineering, Knossos Avenue, University of Crete, Heraklion, GR-71409, Greece; 2 Department of Biology, School of Sciences and Engineering, Vassilika Vouton, University of Crete, Heraklion, GR-70013, Greece; 3 Foundation for Research and Technology – Hellas (FORTH), Institute of Molecular Biology and Biotechnology (IMBB), Heraklion, GR-70013, Greece; 4 Leibniz Institute for the Analysis of Biodiversity Change, Museum Koenig Bonn, Adenauerallee 127, Bonn, 53113, Germany; 5 Metazoa Phylogenomics Lab, Passeig marítim de la Barceloneta 37-49., Institute for Evolutionary Biology (CSIC-UPF), Barcelona, 08003, Spain; 6 Genoscope, Institut François Jacob, CEA, CNRS, Univ Evry, Université Paris-Saclay, Evry, 91057, France; 7 Génomique Métabolique, Genoscope, Institut François Jacob, CEA, CNRS, Univ Evry, Université Paris-Saclay, Evry, 91057, France; 8 European Molecular Biology Laboratory, European Bioinformatics Institute, Wellcome Genome Campus, Hinxton, Cambridge, CB10 1SD, UK; 9 SIB Swiss Institute of Bioinformatics, Amphipôle, Quartier UNIL-Sorge, Lausanne, 1015, Switzerland

**Keywords:** Pipistrellus hanaki, genome assembly, European Reference Genome Atlas, Biodiversity Genomics Europe, Earth Biogenome Project, Vespertilionidae family, Hanak's bat, Νανονυχτερίδα του Hanak

## Abstract

Hanak's bat ( *Pipistrellus hanaki* Hulva and Benda 2004) is one of the most range restricted mammals in Europe, since it occurs only in Cyrenaica, Libya, and Crete (Greece). It is currently classified as 'Vulnerable' on the IUCN Red List, with its foraging habitat threatened by a number of human activities. The reference genome of Hanak's bat ( *Pipistrellus hanaki*) will provide a crucial resource for uncovering the species phylogenetic history and will help assess the degree of genetic isolation among its populations. A total of 23 contiguous chromosomal pseudomolecules (sex chromosomes included) were assembled from the genome sequence. This chromosome-level assembly encompasses 1.9 Gb, composed of 447 contigs and 141 scaffolds, with contig and scaffold N50 values of 48.7 Mb and 89.1 Mb, respectively.

## Introduction

**Figure 1.**
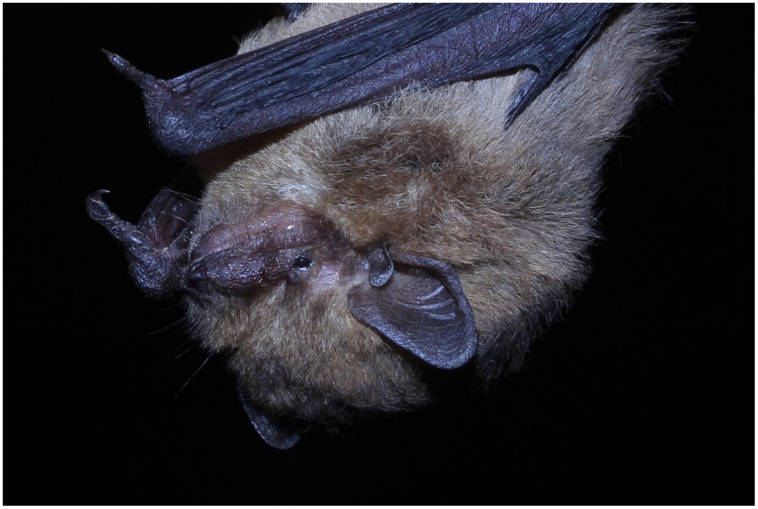
A specimen of the species *Pipistrellus hanaki*. The individual in the picture is a different specimen from the one sampled and sequenced for this study. Photo credit: Panagiotis GEORGIAKAKIS, Natural History Museum of Crete, Crete.

Hanak's bat ( *Pipistrellus hanaki* Hulva and Benda 2004) or Νανονυχτερίδα του Hanak in Greek, is one of the six west-Palaearctic members of the genus *Pipistrellus* Kaup, 1829 s.str ( Figure 1). Phylogenetically, it is the sister species to *Pipistrellus pygmaeus* (Leach, 1825). Hanak's bat is a small bat, with a pale brown to rusty brown pelage coloration, moderately paler and distinctly rustier than in the other two congeneric species, *Pipistrellus pipistrellus* and *Pipistrellus pygmaeus*. The face, wing membranes, ears, and tragi are dark brown, whereas the ear bases and the area around the eyes are slightly paler. In Europe, only a small-sized form of Hanak's bat occurs in Crete, *P. hanaki creticus* Benda, 2009, while the nominotypical form, *P. hanaki hanaki* Hulva and Benda, 2004 lives just in northern Cyrenaica, Libya. These two forms differ in morphometric and genetic traits ( Benda *et al.*, 2004; Benda *et al.*, 2008; Benda *et al.*, 2014); while *P. hanaki hanaki* is the largest member of the common pipistrelle ( *Pipistrellus pipistrellus*) group, *P. hanaki creticus* has a significant smaller body and skull size and its endemic Cretan population was even suggested to represent a separate species, since its phylogenetic position was not fully resolved, but it was shown to be a sister species to both *P. hanaki* s.str and *P. pygmaeus* ( Benda *et al.*, 2014). This phylogenetic reconstruction, however, has not yet been accepted.

The Hanak's bat was first discovered in the north-eastern part of Cyrenaica, Libya ( Benda *et al.*, 2004) and was subsequently found solely in the center of the forested northern part of the Cyrenaican plateau (Jebel Al Akhdar Mts). Its presence on Crete was first documented in 2007 ( Hulva *et al.*, 2007) from a specimen collected in the western part of the island which was analysed morphologically and genetically. Since then, it has been found in several localities, mostly in forested areas, tree cultivations, and vegetated wetlands of western and central Crete ( Benda *et al.*, 2008; Georgiakakis *et al.*, 2023). Although rather widespread on Crete, *Pipistrellus hanaki* was not located in several sampling efforts that were recently undertaken in many islands of south-east Greece, viz. Rhodes and Karpathos ( Georgiakakis *et al.*, 2023).

*Pipistrellus hanaki* is currently classified as 'Vulnerable' on the IUCN Red List ( Georgiakakis *et al.*, 2020), reflecting its high risk of extinction in the wild because of, among others, significant habitat loss due to residential and commercial development, forest fires and expansion of cultivations ( Georgiakakis *et al.*, 2018). Additionally, it is listed under Annex IV of the Habitats Directive (92/43/EU) and Appendix II of the Bern Convention highlighting its protection and conservation status as a species of European interest and necessitating the proper management of its habitat.

*Pipistrellus hanaki* utilizes a great variety of roosts (trees, rock fissures, buildings), but it depends on mature trees – native *Quercus* but also cultivated *Olea*, *Ceratonia*, *Ficus* and *Prunus* – for foraging ( Georgiakakis *et al.*, 2018). As an insect predator, it plays an important role in the forest ecosystem function and resilience.

Developing a high-quality reference genome for *Pipistrellus hanaki* is essential to advance our understanding of its unique genetic makeup. This genomic resource will also support conservation efforts by providing valuable insights into its phylogenetic relationships within the *P. pipistrellus* group and the genetic differences between *P. hanaki hanaki* and *P. hanaki creticus*.

The generation of this reference resource was coordinated by the European Reference Genome Atlas (ERGA) initiative’s Biodiversity Genomics Europe (BGE) project, supporting ERGA’s aims of promoting transnational cooperation to promote advances in the application of genomics technologies to protect and restore biodiversity ( Mazzoni *et al.*, 2023).

## Materials & methods

ERGA's sequencing strategy includes Oxford Nanopore Technology (ONT) and/or Pacific Biosciences (PacBio) for long-read sequencing, along with Hi-C sequencing for chromosomal architecture, Illumina Paired-End (PE) for polishing (i.e. recommended for ONT-only assemblies), and RNA sequencing for transcriptome profiling, to facilitate genome assembly and annotation.

### Sample and sampling information

On 21 October 2023, one adult male of *Pipistrellus hanaki* was sampled by Panagiotis Georgakakis from the Natural History Museum of Crete (NHMC) of the University of Crete. The species was identified using the identification key of Dietz & Kiefer (2016). The specimen was collected with a mist nest in Psiloritis mt., Rouvas forest, Irakleio, Crete, Greece. Sampling was performed under Presidential Decree 67/1981 issued by the Greek Government. The specimen was euthanized by increasing concentration of CO _2_, after which it was immediately flash-frozen in liquid nitrogen, and preserved at -80 °C until DNA extraction.

### Vouchering information

Physical reference material for the here sequenced specimen has been deposited in the Vertebrates Collections of the NHMC https://www.nhmc.uoc.gr/en/departments/vertebrates under accession ID NHMC.80.5.121.52.

Frozen reference tissue material of muscle and liver is available from the same individual at the Genomics and Genetic Resources Division of the NHMC https://www.nhmc.uoc.gr/en/departments/genomics under accession ID NHMC.80.5.121.52.

### Genetic information

The estimated genome size, estimated by Genomes on a Tree (GoaT) ( Challis *et al.*, 2023) by ancestral state reconstruction, is 2.12 Gb. This is a diploid genome with a haploid number of 22 chromosomes (2n=44). All information for this species was retrieved from GoaT.

### DNA/RNA processing

DNA was extracted from muscle (45 mg) using a Genomic-tip 100/G Kit (QIAGEN, MD, USA) following manufacturer instructions. DNA fragment size selection was performed using Short Read Eliminator (PacBio, CA, USA). Quantification was performed using a Qubit dsDNA HS Assay kit (Thermo Fisher Scientific) and integrity was assessed in a FemtoPulse system (Agilent). DNA was stored at 4 °C until usage.

RNA was extracted from 10 mg of muscle using the RNeasy Plus Universal kit (Qiagen) following manufacturer instructions. Residual genomic DNA was removed with 6U of TURBO DNase (2 U/μL) (Thermo Fisher Scientific). Quantification was performed using a Qubit RNA HS Assay kit and integrity was assessed in a Bioanalyzer system (Agilent). RNA was stored at -80 °C.

### Library preparation and sequencing

Long-read DNA libraries were prepared with the SMRTbell prep kit 3.0 following manufacturers' instructions and sequenced on a Revio system (PacBio). Hi-C libraries were generated from muscle (20 mg) of the same individual using the Arima High Coverage HiC kit (following the Animal Tissues low input protocol v01) and sequenced on a NovaSeq 6000 instrument (Illumina) with 2x150 bp read length. Poly(A) RNA-Seq libraries were constructed using the Illumina Stranded mRNA Prep, Ligation Prep kit (Illumina) and sequenced on an Illumina NovaSeq X Plus instrument (Illumina) with 2x150 bp read length. In total, 36x PacBio and 19x HiC data were sequenced to generate the assembly.

### Genome assembly methods

The genome of *Pipistrellus hanaki* was assembled using the Genoscope GALOP pipeline ( https://workflowhub.eu/workflows/1200). Briefly, raw PacBio HiFi reads were assembled using Hifiasm v0.19.5-r593 ( Cheng *et al.*, 2021). Remaining allelic duplications were removed using purge_dups v1.2.5 ( Guan *et al.*, 2020) with default parameters and the proposed cutoffs. The purged assembly was scaffolded using YaHS v1.2 ( Zhou *et al.*, 2023) and assembled scaffolds were then curated through manual inspection using PretextView v0.2.5 to remove false joins and incorporate sequences not automatically scaffolded into their respective locations within the chromosomal pseudomolecules. Summary analysis of the released assembly was performed using the ERGA-BGE Genome Report ASM Galaxy workflow ( 10.48546/workflowhub.workflow.1104.1).

### Genome annotation methods

A gene set was generated using the Ensembl Gene Annotation system ( Aken *et al.*, 2016), primarily by aligning short-read RNA-seq data from BioSample SAMEA115120717 to a previous version of the reference genome (GCA_964339955.1). Gaps in the annotation were filled via protein-to-genome alignments of a select set of vertebrate proteins from UniProt ( UniProt Consortium, 2019), which had experimental evidence at the protein or transcript level. At each locus, data were aggregated and consolidated, prioritising models derived from RNA-seq data, resulting in a final set of gene models and associated non-redundant transcript sets. To distinguish true isoforms from fragments, the likelihood of each open reading frame (ORF) was evaluated against known vertebrate proteins. Low-quality transcript models, such as those showing evidence of fragmented ORFs, were removed. In cases where RNA-seq data were fragmented or absent, homology data were prioritised, favouring longer transcripts with strong intron support from short-read data. The resulting gene models were classified into three categories: protein-coding, pseudogene, and long non-coding. Models with hits to known proteins and few structural abnormalities were classified as protein-coding. Models with hits to known proteins but displaying abnormalities, such as the absence of a start codon, non-canonical splicing, unusually small intron structures (<75 bp), or excessive repeat coverage, were reclassified as pseudogenes. Single-exon models with a corresponding multi-exon copy elsewhere in the genome were classified as processed (retrotransposed) pseudogenes. Models that did not fit any of the previously described categories did not overlap protein-coding genes and were constructed from transcriptomic data were considered potential lncRNAs. Potential lncRNAs were further filtered to remove single-exon loci due to their unreliability. Putative miRNAs were predicted by performing a BLAST search of miRBase ( Kozomara *et al.*, 2019) against the genome, followed by RNAfold analysis ( Gruber *et al.*, 2008). Other small non-coding loci were identified by scanning the genome with Rfam ( Kalvari *et al.*, 2018) and passing the results through Infernal ( Nawrocki & Eddy, 2013).

## Results

### Genome assembly

**Figure 2.**
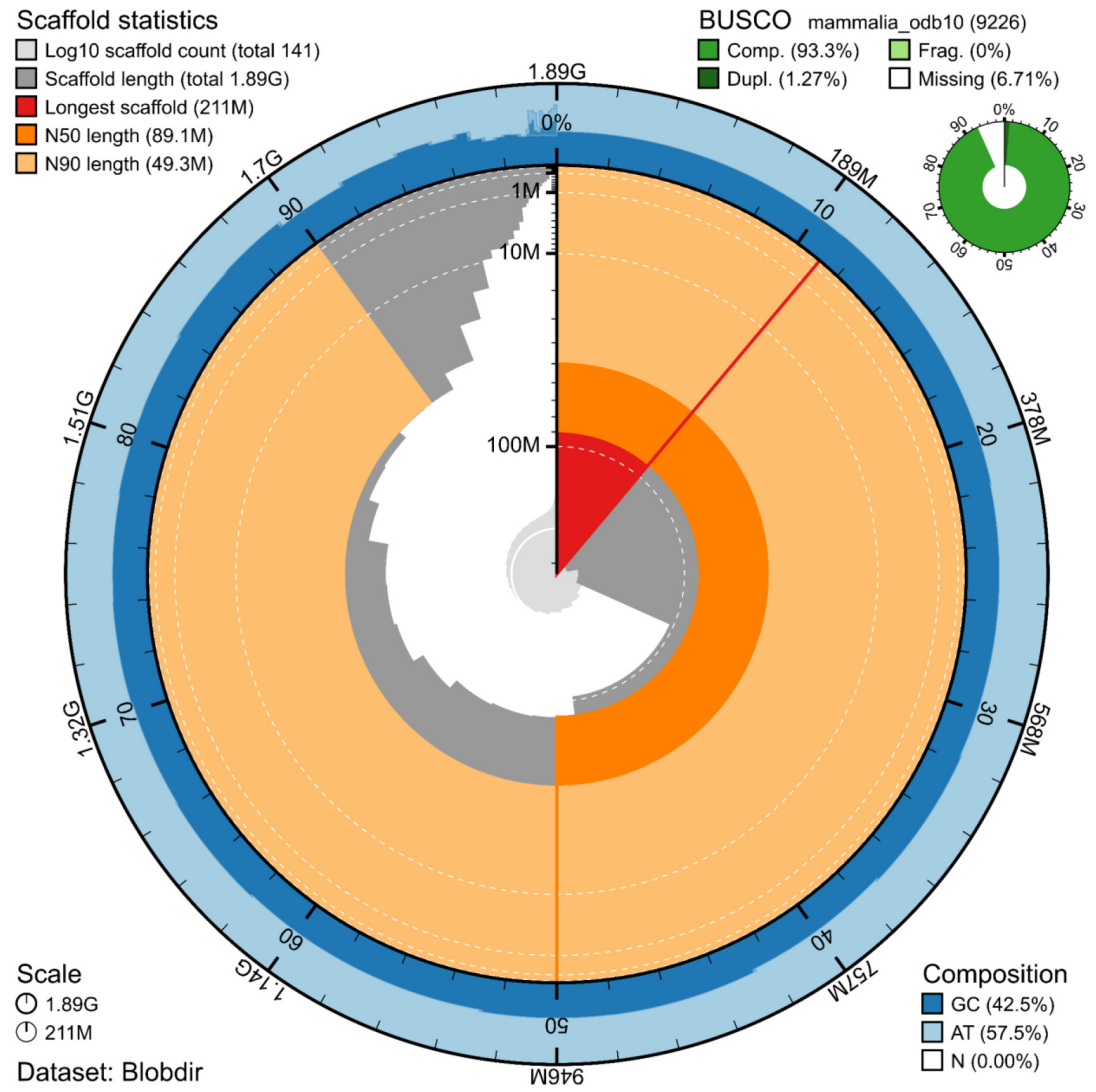
Snail plot summary of assembly statistics. The main plot is divided into 1,000 size-ordered bins around the circumference, with each bin representing 0.1% of the 1,892,181,625 bp assembly. The distribution of sequence lengths is shown in dark grey, with the plot radius scaled to the longest sequence present in the assembly (211 Mb, shown in red). Orange and pale-orange arcs show the scaffold N50 and N90 sequence lengths (89,115,682 and 49,289,172 bp), respectively. The pale grey spiral shows the cumulative sequence count on a log-scale, with white scale lines showing successive orders of magnitude. The blue and pale-blue area around the outside of the plot shows the distribution of GC, AT, and N percentages in the same bins as the inner plot. A summary of complete, fragmented, duplicated, and missing BUSCO genes found in the assembled genome from the Mammalia database (odb10) is shown on the top right.

**Figure 3.**
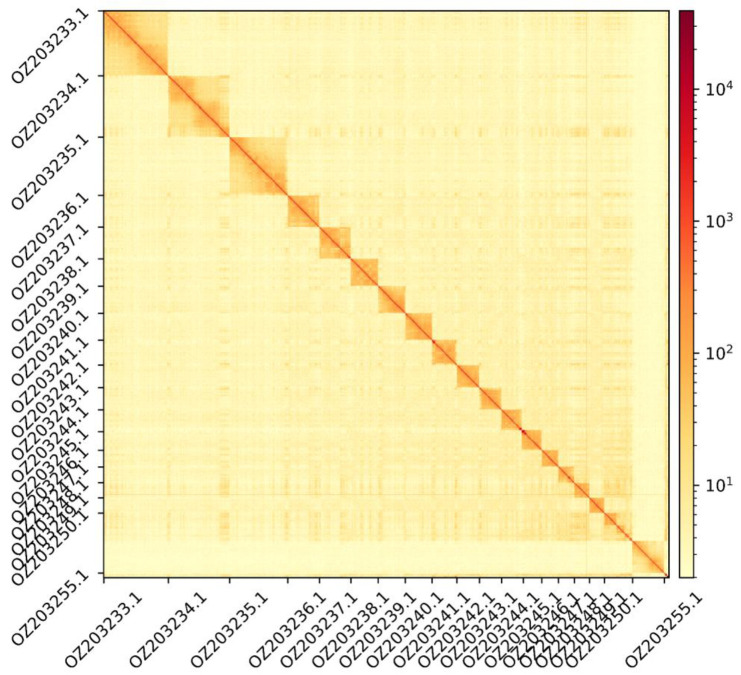
Hi-C contact map showing spatial interactions between regions of the genome. The diagonal corresponds to intra-chromosomal contacts, depicting chromosome boundaries. The frequency of contacts is shown on a logarithmic heatmap scale. Hi-C matrix bins were merged into a 50 kb bin size for plotting. Due to space constraints on the axes, only the GenBank names of the 18th largest autosomes and the Y chromosome (GenBank: OZ203255.1) are shown.

The genome assembly has a total length of 1,892,181,625 bp in 141 scaffolds ( Figure 2 and Figure 3), with a GC content of 42.5%. It features a contig N50 of 48,700,701 bp (L50=16) and a scaffold N50 of 89,115,682 bp (L50=7). There are 306 gaps, totalling 34.1 kb in cumulative size. The single-copy gene content analysis using the Mammalia database with BUSCO ( Manni *et al.*, 2021) resulted in 93.3% completeness (92.0% single and 1.3% duplicated). 96.3% of reads k-mers were present in the assembly and the assembly has a base accuracy Quality Value (QV) of 61.4 as calculated by Merqury ( Rhie *et al.*, 2020).

### Genome annotation

The genome annotation consists of 18,099 protein-coding genes with associated 30,313 transcripts, in addition to 5,633 non-coding genes ( Table 1). Using the longest isoform per transcript, the single-copy gene content analysis using the Mammalia odb10 database with BUSCO resulted in 95.3% completeness. Using the OMAmer Metazoa-v2.0.0.h5 database for OMArk ( Nevers *et al.*, 2025) resulted in 95.7% completeness and 98.5% consistency ( Table 2).

**Table 1.** Statistics from assembled gene models.

	No. genes	No. transcripts	Mean gene length (bp)	No. single- exon genes	Mean exons per transcript
**mRNA**	18,099	30,313	41,209	1,346	11.4
**pseudogene**	386	386	14,593	33	13.3
**snoRNA**	972	972	123	972	1.0
**lncRNA**	3,043	3,204	3,256	2,630	1.4
**miRNA**	135	135	82	135	1.0
**snRNA**	1,263	1,263	119	1,263	1.0
**rRNA**	110	110	225	110	1.0
**scRNA**	77	77	148	77	1.0
**Other ncRNA**	33	33	96-281	33	1.0

**Table 2.** Annotation completeness and consistency scores calculated by BUSCO run in protein mode (mammalia_odb10) and OMArk (Metazoa-v2.0.0.h5).

	Complete	Single copy	Duplicated	Fragmented	Missing
**BUSCO**	8,790 (95.3%)	8,706 (94.4%)	84 (0.9%)	74 (0.8%)	362 (3.9%)
**OMArk**	13,499 (95.7%)	13,207 (93.7%)	292 (2.0%)	-	596 (4.2%)
	Consistent	Inconsistent	Contaminants	Unknown	
**OMArk**	17,942 (98.5%)	197 (1.1%)	0.0 (0.0%)	81 (0.4%)	

## Data Availability

*Pipistrellus hanaki* and the related genomic study were assigned to Tree of Life ID (ToLID) 'mPipHan1' and all sample, sequence, and assembly information are available under the umbrella BioProject PRJEB77247. The sample information is available at the following BioSample accessions: SAMEA115799862, SAMEA115799867, and SAMEA115120717. The genome assembly is accessible from ENA under accession number GCA_964339955.4 and the annotated genome is available at the Ensembl website ( https://projects.ensembl.org/erga-bge/). Sequencing data produced as part of this project are available from ENA at the following accessions: ERX12737184, ERX12737185, ERX14169058, ERX14169059, and ERX12733454. Documentation related to the genome assembly and curation can be found in the ERGA Assembly Report (EAR) document available at https://github.com/ERGA-consortium/EARs/tree/main/Assembly_Reports/Pipistrellus_hanaki/mPipHan1. Further details and data about the project are hosted on the ERGA portal at https://portal.erga-biodiversity.eu/data_portal/412090.

## Author information

Members of the Genoscope Sequencing Team are listed here: https://zenodo.org/records/14611490.
