# Supplementary material for: ERGA-BGE reference genome of the forest-restricted Cretan subspecies of Hanak's bat ( Pipistrellus hanaki creticus), an IUCN Vulnerable species
Source: Open Res Eur. 2026 Apr 11;5:298. Originally published 2025 Sep 25. [Version 3] doi: 10.12688/openreseurope.20937.3 (PMC12631070; doi:10.12688/openreseurope.20937.3)
Supplement: Peer review history

# Reviewer report: Reviewer response for version 2

**DOI:** 10.21956/openreseurope.23670.r63933

**Published:** 2025-11-19

**Authors:** Jiang Feng

**Affiliations:** 1 Northeast Normal University, Changchun, China

This manuscript presents a high-quality chromosome-level reference genome assembly and annotation for *Pipistrellus hanaki*—a IUCN Vulnerable species with a narrow distribution. The study follows standardized genomic workflows, including PacBio HiFi sequencing and Hi-C scaffolding, and provides comprehensive assembly statistics and annotation details, which is valuable for enrich genomic resource of this species and supporting its conservation. There are two issues regarding species taxonomic clarity and methodological rational need to be addressed to enhance the manuscript’s scientific rigor and practical utility.

 1.A critical inconsistency exists between the manuscript’s title and the actual sequenced subspecies. The title refers to Hanak's bat ( *Pipistrellus hanaki*), but the Introduction explicitly states that the sequenced individual is from Crete, belonging to the subspecies *P. hanaki creticus* (not the nominotypical *P. hanaki hanaki* from Libya). Moreover, the manuscript fails to address whether the genome of *P. hanaki creticus *can fully represent the entire species, especially given previous debates about the Cretan population potentially being a distinct species (Benda et al., 2014). This ambiguity undermines the generalizability of the "reference genome" and confuses readers about its taxonomic scope. Therefore, the authors are suggested to revise the title to explicitly reflect the subspecies, e.g., "ERGA-BGE reference genome of the Cretan subspecies of Hanak's bat ( *Pipistrellus hanaki creticus*), an IUCN Vulnerable species restricted to forest-like biotopes". And add a dedicated subsection in the Discussion to address: 1) genetic differences between *P. hanaki creticus* and *P. hanaki hanaki*; 2) limitations of using a single subspecies to represent the entire species, and recommendations for future genome sequencing of the Libyan subspecies to enable inter-subspecies comparisons.

 2. The Methods section states that gene annotation relied on "publicly available short-read RNA-seq data from BioSample SAMEA115120717" aligned to a previous version of the genome (GCA_964339955.1), rather than RNA-seq data generated from the newly assembled genome in this study. No explanation is provided for this choice, why public data was prioritized. This raises concerns about annotation accuracy, as public data may not perfectly match the new assembly or the specific Cretan subspecies. If own RNA-seq data was generated but unused, explain technical issues (low quality? insufficient depth?) and validate the compatibility of the public data with the new assembly (e.g., sequence identity, alignment rate). If no new RNA-seq data was generated, acknowledge this limitation and discuss potential biases (such as public data from a different individual/population affecting gene model accuracy).

 3. Other minor issues

 The phrase "collected with a mist nest" may contain a spelling error. "Mist net" is the standard term for the tool used in bat sampling (to capture flying individuals).

 In Introduction, multiple citations are overly repetitive. For example, "(Benda et al., 2004; Benda et al., 2008; Benda et al., 2014)") should be "(Benda et al., 2004, 2008, 2014)".

Are sufficient details of methods and materials provided to allow replication by others?

Yes

Is the rationale for creating the dataset(s) clearly described?

Yes

Are the datasets clearly presented in a useable and accessible format?

Yes

Are the protocols appropriate and is the work technically sound?

Yes

Reviewer Expertise:

Animal molecular ecology

I confirm that I have read this submission and believe that I have an appropriate level of expertise to confirm that it is of an acceptable scientific standard.

# Response

**Published:** 2026-03-18

**Authors:** Chiara Bortoluzzi

**Affiliations:** Swiss Institute of Bioinformatics, Lausanne, Vaud, Switzerland

We thank the reviewer for their feedback. We address all of their comments as below: 

1. As pointed out by the reviewer, the manuscript now focuses on the Cretan subspecies of Hanak's bat ( *Pipistrellus hanaki creticus*). The manuscript has thus been changed accordingly throughout. 
2. As already addressed for reviewer 2, we rephrased the RNA-Seq data statement to prevent further misunderstanding. Briefly, short-read RNA-Seq data produced for the assembled species were used for the genome annotation. These RNA-Seq data are publicly available. 
3. We changed the sentence on the collection method.
4. We corrected the citations in the introduction.

# Reviewer report: Reviewer response for version 1

**DOI:** 10.21956/openreseurope.22651.r61360

**Published:** 2025-11-03

**Authors:** Richard Orton

**Affiliations:** 1 University of Glasgow, Glasgow, UK

Overall – I found this to be a very well written report. I have only a couple of minor suggestions:

 Introduction – suggestion - a picture of Hanak’s bat might be useful here to go with the description

 “In Europe, only a small-sized form of Hanak's bat occurs in Crete, *P. hanaki creticus* Benda, 2009, while the nominotypical form, *P. hanaki hanaki* Hulva and Benda, 2004 lives just in northern Cyrenaica, Libya. These two forms differ in morphometric and genetic traits ( Benda *et al.*, 2004; Benda *et al.*, 2008; Benda *et al.*, 2014);” - rephrase – presumably the Hulva and Benda inclusions mid sentence are incorrectly formatted refs?

 Overall – is there an expectation that the Crete and Libya populations of Hanak’s bat are the same species and therefore the same genome? The title of the manuscript is Hanak's bat ( *Pipistrellus hanaki*) – but according to the sentence highlighted in the previous point you are sequencing *P. hanaki criticus. *Maybe a sentence somewhere in discussion to address?

 “A gene set was generated using the Ensembl Gene Annotation system ( Aken *et al.*, 2016), primarily by aligning publicly available short-read RNA-seq data from BioSample SAMEA115120717 to a previous version of the reference genome (GCA_964339955.1).” Clarification - unsure what is happening here – you seem to be using public data with an existing reference genome – why are you not using your own one that was created?

 What quality control has been done on the scaffolds/contigs? i.e. often viral transcripts (both true retroviral transcripts which are in then genome, but also erroneous viruses that infected the sequenced host) can be found in provisional genome sequences – have these been checked for (just a suggestion).

Are sufficient details of methods and materials provided to allow replication by others?

Yes

Is the rationale for creating the dataset(s) clearly described?

Yes

Are the datasets clearly presented in a useable and accessible format?

Yes

Are the protocols appropriate and is the work technically sound?

Yes

Reviewer Expertise:

Bioinformatics, Metagenomics, Sequencing

I confirm that I have read this submission and believe that I have an appropriate level of expertise to confirm that it is of an acceptable scientific standard.

# Response

**Published:** 2025-11-07

**Authors:** Chiara Bortoluzzi

**Affiliations:** Swiss Institute of Bioinformatics, Lausanne, Vaud, Switzerland

As suggested by the reviewer, we now present in the Introduction a picture of an Hanak's bat individual. We did not change the formatting of the references when referring to *P. hanaki creticus *and *P. hanaki hanaki, *as we follow here standard international nomenclature rules. We rephrased the section on the gene annotation to avoid any further misunderstanding about the RNA-seq data used in the gene annotation step. We also would like to refer the reviewer to the EAR report (we refer to this in the main text of this Genome Report), as this report provides a comprehensive overview of all contamination analyses performed during the assembly step.
